# PLK1 Down-Regulates Parainfluenza Virus 5 Gene Expression

**DOI:** 10.1371/journal.ppat.1000525

**Published:** 2009-07-24

**Authors:** Dengyun Sun, Priya Luthra, Zhuo Li, Biao He

**Affiliations:** 1 Intercollege Graduate Program in Cell and Developmental Biology, Pennsylvania State University, University Park, Pennsylvania, United States of America; 2 Department of Veterinary and Biomedical Sciences, Pennsylvania State University, University Park, Pennsylvania, United States of America; 3 Center of Molecular Immunology and Infectious Disease, Pennsylvania State University, University Park, Pennsylvania, United States of America; Mount Sinai School of Medicine, United States of America

## Abstract

The paramyxoviruses are a family of negative-sense RNA viruses that includes many important human and animal pathogens. Paramyxovirus RNA synthesis requires the viral phosphoprotein (P) and the large (L) protein. Phosphorylation of P is thought to regulate viral gene expression, though direct proof remains elusive. Recently, we reported that phosphorylation of a specific residue (Ser157) of the P protein of parainfluenza virus 5 (PIV5), a prototypical paramyxovirus, correlates with decreased viral gene expression and cytokine expression in infected cells. Here, we show that: Polo-like kinase 1 (PLK1), a serine/theronine kinase that plays a critical role in regulating the cell cycle, interacts with PIV5 P through the S157 residue; PLK1 inhibition increases viral gene expression; PLK1 over-expression inhibits viral gene expression; and PLK1 directly phosphorylates P *in vitro*, indicating that PLK1 down-regulates viral gene expression by phosphorylating P. Furthermore, we have determined the PLK1 phosphorylation site on P and found that mutant recombinant PIV5 whose P proteins cannot either bind to or be phosphorylated by PLK1 have similar phenotypes. Increased viral gene expression in PIV5 with mutations in the PLK1 binding/phosphorylation sites correlates with increased induction of cell death and cytokine expression, suggesting that PIV5 limits its viral gene expression to avoid these host effects. It is possible that targeting PLK1 will enhance host innate immune responses, leading to a novel strategy of clearing paramyxovirus infections quickly.

## Author Summary

The paramyxoviruses are a family of negative-sense RNA viruses that includes many important human and animal pathogens. Paramyxovirus RNA synthesis requires the viral phosphoprotein (P) and the large (L) protein. Phosphorylation of P is thought to regulate viral gene expression, though direct proof remains elusive. Here, we show that Polo-like kinase 1 (PLK1) interacts with the P protein of parainfluenza virus 5 (PIV5), a prototypical paramyxovirus, through the serine residue at position 157 and phosphorylates P at serine residue 308. Inhibition of PLK1 increases viral gene expression. Increased viral gene expression in cells infected by PIV5 with mutations in the PLK1 binding/phosphorylation sites correlates with increased induction of cell death and cytokine expression, suggesting that PIV5 limits its gene expression to avoid induction of innate immune responses. It is possible that targeting PLK1 will enhance host innate immune responses, leading to a novel strategy of anti-paramyxovirus therapy.

## Introduction

Viruses in the *Paramyxoviridae* family of *Mononegavirales* include many important human and animal pathogens such as the human parainfluenza viruses, Sendai virus (SeV), mumps virus (MuV), Newcastle disease virus (NDV), measles virus (MeV), rinderpest virus and human respiratory syncytial virus (RSV) as well as the emerging viruses Nipah and Hendra virus. The paramyxovirus RNA-dependent RNA polymerase (RdRp), which both transcribes and replicates the viral RNA genome, consists of two proteins, the phosphoprotein (P) and the large (L) protein [1]. While paramyxovirus P proteins are all heavily phosphorylated (hence the name phosphoprotein) and are essential for viral gene expression, the role of P phosphorylation in the replication of paramyxoviruses remains an enigma. Conclusive evidence on the role of phosphorylation of the P protein in replication of paramyxoviruses remains elusive. The most recent work seems to indicate that the phosphorylation of the P proteins of paramyxoviruses does not have a role in viral gene expression. The best-studied P proteins of paramyxoviruses are the P proteins of RSV and SeV. It was first reported in the 1970s that the P protein of SeV is phosphorylated [2]. While as many as 11 phosphorylation sites were detected, the serine (Ser) residue at position 249 was determined to be the major phosphorylation site [3]. However, recombinant SeV containing mutations at the major P phosphorylation sites have similar growth characteristics and pathogenicity *in vitro* (cultured cells) and *in vivo* (mice) [4], indicating that these sites are not important for viral gene expression. Mutating five additional phosphorylation sites besides S249 results in a P mutant whose level of phosphorylation is reduced by more than 90% in transfected cells; yet, the mutant P still has normal activity in a mini-genome system [5]. The P protein of RSV is the most heavily phosphorylated of the paramyxovirus P proteins [6]. Two clusters of phosphorylation sites (amino acid residues 116, 117 and 119 and residues 232 and 237) have been identified [7]–[10]. When mutations are introduced into these sites in recombinant RSV by a reverse genetics system, expression levels of the viral genes are not adversely affected, indicating that these residues do not play a critical role in viral gene expression [11]. Further studies of the P protein using mass spectrometry identified the threonine residue at position 108 as being phosphorylated. The phosphorylation of T108 is important for its interaction with M2-1, a processivity factor of viral RNA synthesis, and mutating this residue results in diminished activity in a mini-genome system, suggesting that P may regulate viral RNA synthesis through its interaction with M2-1 [12]. However, the role of this phosphorylation site has not been examined in the context of virus infection. The P protein of HPIV3 is phosphorylated by protein kinase C isoform ζ (PKC-ζ) [13] and the serine residue at position 333 is the likely target site [14]. However, the role of phosphorylation at Ser 333 in the virus life cycle has not been reported. Thus, to the best of our knowledge, regulation of paramyxovirus viral gene expression by phosphorylation state of P has never been directly demonstrated in virus-infected cells even though it is thought that the phosphorylation of the P protein is critical for its function in viral gene expression.

**Figure 1:**
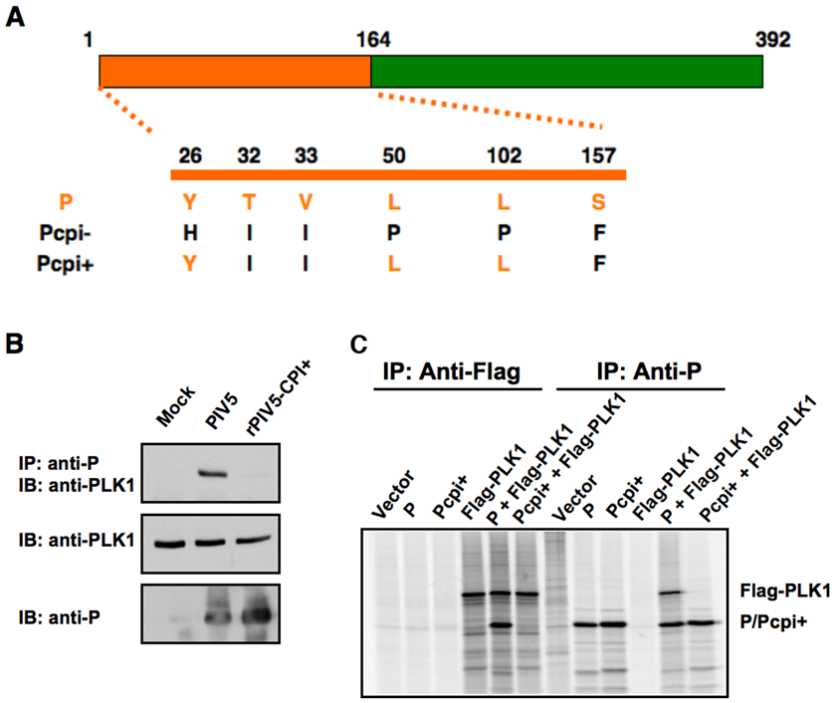
Interaction between P and PLK1. (A). Amino acid residue differences among N-termini of wild type P, Pcpi− and Pcpi+. (B). Interaction between P and endogenous PLK1 in infected cells. HeLa cells were infected with mock, PIV5 or rPIV5-CPI+ and immunoprecipitated (IP) with anti-P antibody. The immunoprecipitated proteins were resolved in SDS-PAGE and subjected to immunoblotting (IB) with antibody against PLK1. (C). Interaction between P and PLK1 in transfected cells. BSR T7 cells were transfected with plasmids encoding P or Pcpi+ along with Flag-PLK1. Vector (pCAGGS) was used to keep the total amount of transfected plasmids constant. At 18–20 hours post transfection, the transfected cells were metabolically labeled with ^35^S-Cys/Met for 3 h at 37°C. The cells were lysed by RIPA buffer and immunoprecipitated with Pk antibody or Flag antibody. The precipitated proteins were resolved by SDS-PAGE gel and visualized using a PhosphorImager.

PIV5, formerly known as simian virus 5 (SV5) [15], is a prototypical paramyxovirus of the *Rubulavirus* genus of the family *Paramyxoviridae* [1]. The PIV5 genome encodes seven genes, from which eight viral proteins are made [1]. The nucleocapsid protein (NP), phosphoprotein (P) and large RNA polymerase (L) protein are essential for viral RNA synthesis (mRNA transcription and genome RNA replication). The V protein plays important roles in viral pathogenesis. The V/P gene of PIV5 is transcribed into both the V mRNA and the P mRNA through a process of pseudo-templated addition of nucleotides, in which the V mRNA is made by faithful transcription the V/P gene and the P mRNA is made by co-transcriptional insertion of two non-templated G residues at a specific site. As a result, the V/P gene is transcribed into two mRNAs and translated into two proteins, which share identical N-termini (164 amino acid residues) but different C-termini. Previously, a strain of PIV5, called canine parainfluenza virus (CPI+), that causes a neurological disorder in canines was isolated [16]. During the course of studying the CPI+ virus, a derivative of CPI+, termed CPI−, was isolated from a dog that was experimentally infected with the CPI+ virus [17]. Several differences were found among the genomes of CPI+, CPI− and the commonly used lab strain, W3A, including in the shared region of the V/P gene [15],[18],[19]. The CPI− virus has eight amino acid residues in the V/P gene that are different from the W3A strain (referred to as wild type in this work) and six of them are in the shared region of the V and the P proteins (the N-terminus 164 amino acid residues). The CPI+ virus has five amino acid residues different from wild type PIV5 in the V/P gene and three of them are in the shared region of the V and P proteins (Fig. 1A). A recombinant PIV5 (W3A strain) containing the CPI− mutations in the shared V and P proteins (rPIV5-CPI−) was shown to have elevated viral gene expression compared to the wild type W3A strain and to induce expression of host anti-viral response genes, such as interferon (IFN)-β and interleukin (IL)-6, and apoptosis in infected cells [20]. Our recently work indicates that a single amino acid residue of P, the serine at position 157 (S157), is responsible for the increased viral gene expression. S157 is phosphorylated and its phosphorylation correlates with decreased viral gene expression as well as the lack of induction of cytokine expression in infected cells [21], suggesting that phosphorylation at S157 plays a role in regulating viral gene expression. However, mechanism of this regulation is not clear.

Intriguingly, S157 residue of P is within a consensus binding motif for Polo-like kinase 1 (PLK1), a serine/theronine kinase that plays a critical role in regulating the cell cycle [22]. PLK1 contains two domains, a polo-box domain (PBD) and a kinase activity domain. It is known that the PBD domain of PLK1 binds to its target through a SpS(orT)P motif (SSP motif), where the second amino acid residue, S or T, is phosphorylated for optimal binding [23]. The binding of PLK1 to a target protein with an SSP motif enables PLK1 to phosphorylate either the target itself or a protein associated with the target [24]. Here, we have investigated the role of PLK1 in the increased viral gene expression of rPIV5-CPI−.

## Results

### PLK1 interacts with P in both infected and transfected cells via the SSP motif

Previously, it has been reported that rPIV5-CPI+, a recombinant virus containing three amino acid changes (V32I, T33I, and S157F) compared to wild type has elevated viral gene expression levels and that S157F is responsible for the phenotype [21]. To ascertain the mechanism of the increased viral gene expression, the amino acid sequence of P was searched for known motifs. S157 is in a PLK1 binding motif [23],[25], suggesting that PLK1 may play a role in regulating viral gene expression. To investigate a possible role of PLK1 in viral gene expression, we examined the interaction between PLK1 and P. HeLa cells were mock-infected or infected with PIV5 or rPIV5-CPI+. The cell lysates were immunoprecipitated with anti-P antibody and then immunoblotted with anti-P or anti-PLK1 antibody. As shown in Fig. 1B, PIV5 P protein interacted with endogenous PLK1, while P protein of rPIV5-CPI+ (Pcpi+) did not. Since P interacts with viral proteins NP and L, we wanted to determine whether PLK1 interacts directly with P. Therefore, we examined the interaction between PLK1 and P in transfected cells. As shown in Fig. 1C, PLK1 was co-immunoprecipitated with P, but not with Pcpi+ by antibody against P; the reciprocal immunoprecipitation with anti-Flag antibody confirmed the interaction of PLK1 with P and not Pcpi+, indicating that PLK1 interacts with P, likely through the SSP motif centered at the residue S157. Furthermore, we carried out the same experiments using immunoprecipitation followed by immunoblotting and obtained the same results (supplemental Fig. S1).

### PLK1 down-regulates PIV5, but not rPIV5-CPi+ gene expression

**Figure 2:**
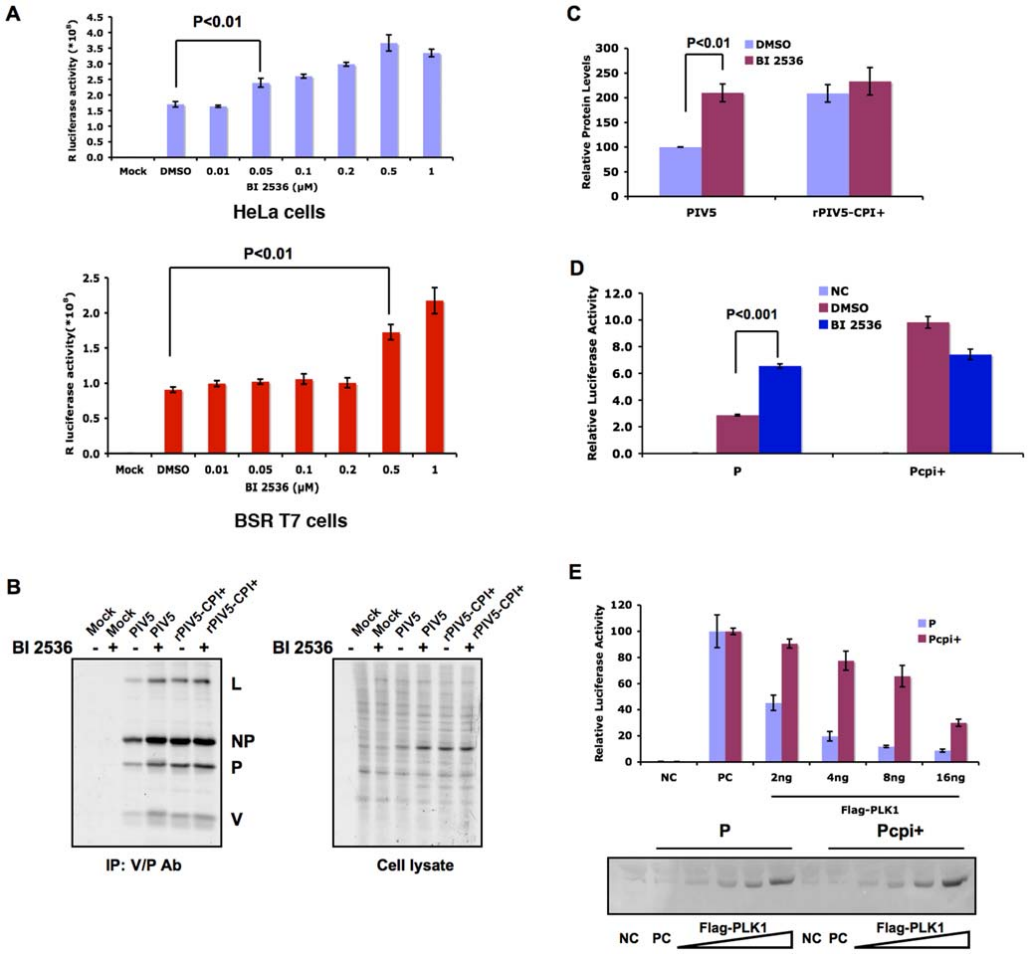
Effects of PLK1 inhibitor and over-expression of PLK1 on viral gene expression. (A). Effect of PLK1 inhibitor BI 2536 on recombinant PIV5 expressing *Renilla* luciferase (rPIV5-RL). HeLa or BSR T7 cells in 24-well plates were infected with rPIV5-RL at MOI of 1 and incubated with BI 2536 at concentration of 0.01, 0.05, 0.1, 0.2, 0.5 and 1 µM. *Renilla* luciferase activity was measured at 18–20 hours post infection and average luciferase activity +/− standard derivation of mean (SEM) is shown. (B). Effect of PLK1 inhibitor BI 2536 on viral gene expression. HeLa cells were mock-infected or infected with PIV5 or rPIV5-CPI+ and incubated in 1 µM BI 2536. The cells were metabolically labeled with ^35^S-Met/Cys at 18–20 hours after infection and immunoprecipitated with Pk antibody, which recognizes V and P and co-precipitates NP and L. The right panel is labeled cell lysates without immunoprecipitation. (C). Quantification of Figure 2B. Six independent experiments were performed and the relative amount of the P protein was measured. The level of PIV5 P in infected cells with DMSO treatment was set at 100 and the rest was normalized to the level of PIV5 P. The average of relative expression level of P +/− SEM is shown. (D). Effects of PLK1 inhibitor on the mini-genome system. A mini-genome plasmid (pSMG-RL) that contains a *Renilla* luciferase (RL) reporter gene and from which a negative-sense mini-genome is generated from T7 RNA polymerase transcription in BSR T7 cells was described previously [21]. In the presence of NP and L and P or P from rPIV5-CPI+ (Pcpi+), this negative-sense RNA template is replicated and transcribed to give rise to the RL mRNA, resulting in luciferase activity. The pT7-FF-Luc plasmid, which contains an FF-Luc reporter gene as a transfection efficiency control, was transfected along with the plasmids. Firefly and *Renilla* luciferase activities were detected in cell lysates at 18 to 20 h post-transfection, as described in Experimental Procedures. BSR T7 cells were transfected with necessary plasmids and incubated with 0.25 µM BI 2536. At 18–20 hours post transfection, the cells were lysed and dual luciferase assay was performed. The relative luciferase activities are calculated at ratios of *Renilla* luciferase activity (indicative of mini-genome replication) versus FF-Luc activity (indicative of transfection efficiency). The average of relative luciferase activity +/− SEM is shown. (E). Effects of PLK1 overexpression on the mini-genome system. Plasmids encoding pCAGGS-Flag-PLK1 at various concentrations were transfected along with the mini-genome system as above. Dual luciferase assay was carried as described above. An aliquot of cell lysate was used for immunoblotting to detect expression levels of Flag-PLK1 (the bottom panel).

To investigate the role of PLK1 in PIV5 gene expression, we tested the effect of a PLK1 inhibitor (BI 2536) on reporter gene expression using a recombinant PIV5 expressing *Renilla* luciferase, (rPIV5-RL). As shown in Fig. 2A, BI 2536 at 0.05 and 1 µM increased *Renilla* luciferase activity in HeLa cells. In BSR T7 cells, BI2536 was also effective in increasing the luciferase activity, albeit at higher concentrations. Because of concerns over potential off-target effects of this molecule, we also tested the effect of a structurally different PLK1 inhibitor, GW843682 (Sigma), in HeLa cells and found the same effect on reporter gene expression as observed with BI 2536 (supplemental Fig. S2). Furthermore, we examined the effect of PLK1 inhibition on viral protein expression. HeLa cells were infected and then metabolically labeled with ^35^S-Cys/Met. The infected cells were immunoprecipitated with antibody (Pk) that recognizes both the P and V protein. Compared with control, BI 2536 treatment increased PIV5 viral protein expression (Fig. 2B) (NP and L bind to P and thus were co-immunoprecipitated with this antibody). In contrast, BI 2536 treatment did not greatly affect cellular protein expression, as shown in the right panel in Fig. 2B, indicating that PLK1 regulation of gene expression in infected cells is specific to PIV5. Interestingly, BI 2536 did not increase viral protein expression in rPIV5-CPI+-infected cells (Fig. 2C), suggesting that PLK1 does not play a role in regulating viral gene expression of rPIV5-CPI+. These results suggest that the SSP motif plays a critical role in the inhibition of viral gene expression by PLK1. To further examine the role of PLK1 in viral gene expression, we used a mini-genome system, in which viral gene expression can be examined free of viral infection [21]. As shown in Fig. 2D, PLK1 inhibitor BI 2536 increased reporter gene activity from the PIV5 mini-genome using wt P, but had little effect on luciferase expression driven by Pcpi+. This result is consistent with the observation in infected cells. We also attempted to utilize siRNA technology to knock down expression of endogenous PLK1 in HeLa cells (data not shown) to examine the effect PLK1 depletion on viral gene expression. Unfortunately, the experiment was inconclusive due to the toxicity of siRNA against PLK1. Optimal knock down of PLK1 expression occurs between 48 to 96 hours after transfection of siRNA, after which we infected the siRNA-treated cells. Since PLK1 plays a critical role in cell cycle progression, this long period of PLK1 depletion, coupled with viral infection, is overwhelmingly cytotoxic.

Since phosphorylation of S157 reduces activity of the P protein, we reasoned that increased phosphorylation of the residue by PLK1 overexpression would further reduce the activity of P. When PLK1 was ectopically expressed in the mini-genome system, PLK1 transfection reduced the mini-genome activity in a dose dependent manner (Fig. 2E): there was about 50% reduction of luciferase activity using 2 ng, and 95% reduction with 16 ng, of PLK1 plasmid in the transfection. PLK1 overexpression had a much weaker effect on mini-genome transcription when Pcpi+ was used in place of wild type P; PLK1 plasmid at 4 ng, 8 ng and 16 ng moderately inhibited the Pcpi+ mini-genome activity, while there was no significant effect at 2 ng though expression levels of PLK1 correlated with amount of plasmids transfected. To confirm that PLK1 binds the SSP motif, we performed similar experiments using P containing a single point mutation S157A instead of Pcpi+, which has three point mutations (V32I, T33I, and S157F). As expected, P-S157A failed to bind PLK1 (supplemental Fig. S3A) and the mini-genome activity using P-S157A was weakly affected by PLK1 over-expression (supplemental Fig. S3B). Those data indicate that PLK1 binds to PIV5 P protein through the SSP motif centered at the residue S157 and reduces PIV5 gene expression. The moderate inhibitory effect of over-expressed PLK1 on Pcpi+ or P-S157A mini-genome system is likely due to an interaction between PLK1 and an additional SS(T)P sequence centered at T108 of P. This STP sequence is not known to be phosphorylated and mutating this T to A had no effect on PLK1 binding or viral gene expression (data not shown). However, since even unphosphorylated STP can weakly interact with PLK1, overexpression of PLK1 may result in interaction of PLK1 (a low level of Pcpi+ and PLK1 was indeed detected when PLK1 was overexpressed in supplemental Fig. S1), resulting in the moderate inhibitory effect.

**Figure 3:**
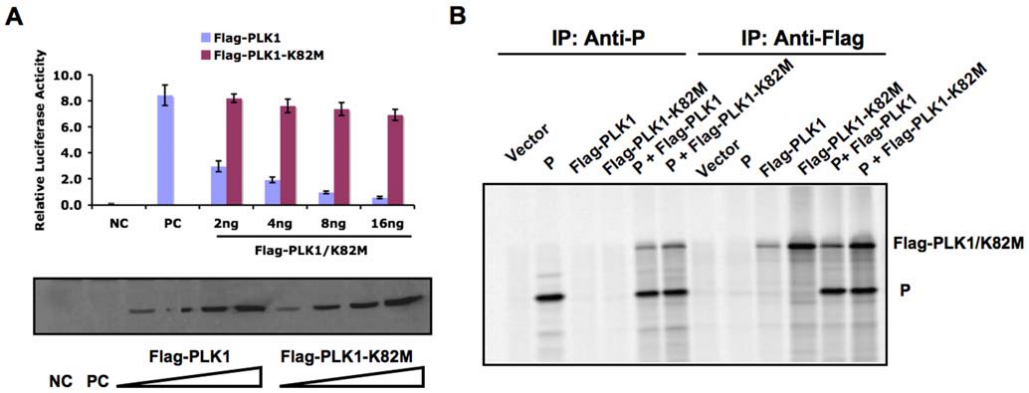
Effect of kinase-deficient PLK1 on PIV5 protein expression. (A). Effects of overexpression of PLK1 kinase-dead mutant K82M on the mini-genome system. BSR T7 cells in 24-well plates were transfected with various concentrations of pCAGGS-Flag-PLK1 or pCAGGS-Flag-PLK1-K82M together with the plasmids necessary for the PIV5 mini-genome system. Dual luciferase assay was carried out after 18–20 h post transfection as before. NC: negative control, no plasmid encoding P; PC: positive control, no plasmid encoding PLK1 or PLK1 K82M. The relative activity unit of positive control was set as 100. The average of relative luciferase activity +/− SEM is shown. An aliquot of cell lysates was used for immunoblotting to detect the expression level of Flag-PLK1 or Flag-PLK1-K82M. (B). Interaction between P and PLK1-K82M. A plasmid encoding P was transfected into cells with a plasmid encoding Flag-PLK1 or Flag-PLK1-K82M. The cells were metabolically labeled and immunoprecipitated with anti-P or anti-Flag.

### Kinase activity of PLK1 is required for its inhibitory effect on PIV5 gene expression

To examine whether the binding of PLK1 is sufficient for its inhibitory effect on PIV5 gene expression, or if its kinase activity is required, we tested a the effect of overexpression of a kinase-deficient PLK1 (PLK1-K82M), which has a lethal point mutation at the ATP binding site (K82M), on PIV5 minigenome activity [26]. As shown in Fig. 3A, PLK1-K82M, at amounts from 2 to 16 ng, failed to inhibit PIV5 mini-genome activity, in contrast to wt PLK1, even though PLK1 and PLK1-K82M were expressed at similar levels, indicating that kinase activity is required for PLK1's inhibitory effect on PIV5 gene expression. Since the binding between PLK1 and target protein is through the PBD domain at the C-terminus of PLK1, we expected that PLK1-K82M would still bind the P protein and this was confirmed by co-immunoprecipitation (Fig. 3B). Thus, the kinase activity of PLK1 is required for PLK1's effect on PIV5 gene expression, while the binding itself is not sufficient.

### PLK1 phosphorylates the P protein in infected cells

**Figure 4:**
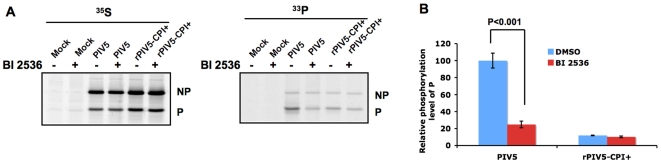
Effects of PLK1 inhibitor on phosphorylation of P. (A). Effects of BI 2536 on phosphorylation of P. Cells were mock-infected or infected with PIV5 or rPIV5-CPI+. At 18–20 hours post infection, the cells were metabolically labeled with ^35^S-Cys/Met or ^33^P-orthophosphate in the presence of 1 µM PLK1 inhibitor BI 2536 or DMSO as described in Experimental Procedures. The cells were lysed and immunoprecipitated with anti-P Pk antibody. (B). Quantification of effects of BI 2536 on phosphorylation of P. Three individual experiments as described in (A) were quantified. The average ratio of phosphorylated P from the ^33^P-labeling experiment to total amount of P as ^35^S-labeled P in PIV5 infected cells treated with DMSO was set to 100 and the others are normalized to the ratio. The average of relative level of phosphorylation of P +/− SEM is shown.

To investigate the mechanism by which PLK1 regulates PIV5 gene expression by PLK1, it was important to determine the phosphorylation target of PLK1. PLK1 can phosphorylate SSP motif-containing proteins themselves or those proteins associated with the SSP-containing protein. We hypothesized that after binding to the P protein, PLK1 phosphorylates P itself in infected cells. To test this, we compared the level of phosphorylated P protein with or without PLK1 inhibitor BI 2536 treatment in PIV5 or rPIV5-CPI+ infected cells. Consistent with previous studies [21], rPIV5-CPI+ infected cells showed higher levels of viral protein expression than that PIV5 infected cells and Pcpi+ had lower level of phosphorylation than that of PIV5 (Fig. 4A). Because we only treated the infected cells for a short period of time during labeling (4 hours), no significant difference of PIV5 viral protein levels between DMSO and BI 2536 treated cells was detected by ^35^S-Cys/Met labeling. However, there was a significant reduction of phosphorylation of P due to BI 2536 treatment in PIV5 infected cells (Fig. 4B), but not in rPIV5-CPI+ infected cells. Interestingly, while NP was also phosphorylated in infected cells, its phosphorylation was not affected by PLK1 inhibitor treatment, indicating that NP phosphorylation is PLK1-independent even though it interacts with P.

### PLK1 phosphorylates the P protein at S308

**Figure 5:**
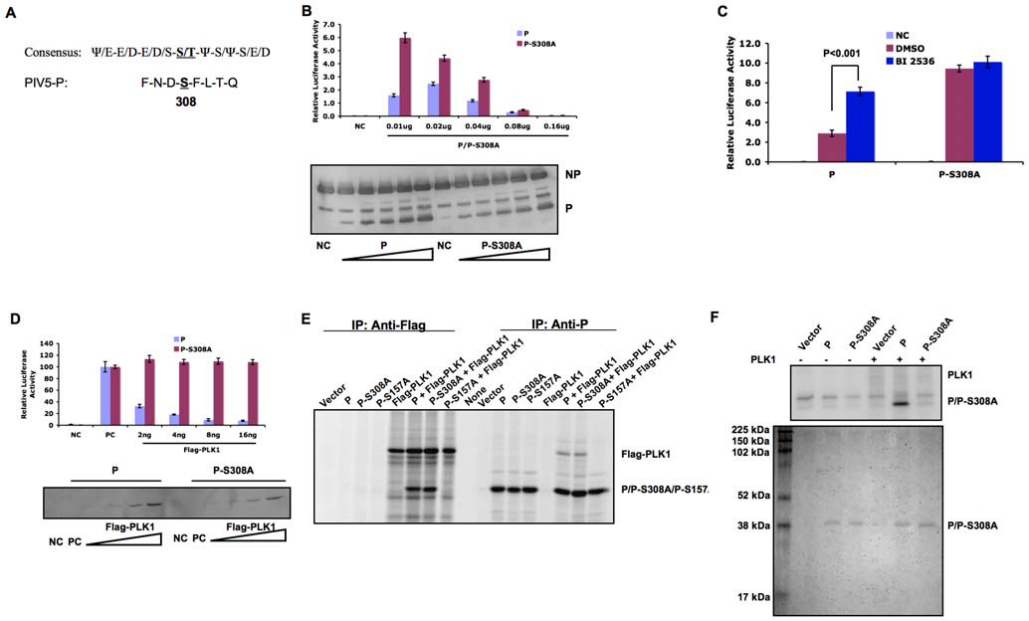
PLK1 phosphorylates the P protein at S308. (A). Prediction of PLK1 phosphorylation site within P protein. The center bold S/T residue is the PLK1 phosphorylation site. X, any amino acid residue; Ψ, amino acid residue with a hydrophobic side chain. (B). Effect of mutating S308 into A on the mini-genome activity. A mini-genome system as described before was used. To ensure a validity comparison of P vs. P-S308A, a range of concentrations of the P proteins in the experiments were used. Cell lysate aliquots of the transfected cells were subjected to immunoblotting using anti-NP or anti-P antibody. The average of relative luciferase activity +/− SEM is shown. (C). Effects of PLK1 inhibitor on the mini-genome system using P-S308A. To study the effect of PLK1 inhibitor on mini-genome activity with mutant P-S308A, BI 2536 was added to the mini-genome system using P-S308A in a similar fashion as in Fig. 2D. (D). Effects of PLK1 overexpression on the mini-genome system using P-S308A. Plasmids encoding pCAGGS-Flag-PLK1 at various concentrations were transfected along with the mini-genome system as above. Dual luciferase assay was carried as described above. An aliquot of cell lysate was used for immunoblotting to detect expression levels of Flag-PLK1. (E). Interaction between P-S308A and PLK1. A plasmid encoding Flag-PLK1 was transfected into cells with a plasmid encoding P or P-S308A. The cells were metabolically labeled and immunoprecipitated with anti-P or anti-Flag. (F). Phosphorylation of P by PLK1 *in vitro*. HeLa cells were transfected with plasmids pCAGGS-P or pCAGGS-P-S308A. P and P-S308A were purified from transfected cells using anti-P as described in Experimental Procedures. Kinase assays were carried out in a total volume of 30 µl containing 100 ng PLK1 for 60 minutes at room temperature. The bottom panel is the input of P and P-S308A with PLK1 on a SDS-PAGE with Coomassie blue staining.

Because PLK1 inhibition reduced P phosphorylation, we wanted to determine the PLK1 phosphorylation site within P. Since a consensus PLK1 phosphorylation site has been reported [27], we searched the sequence of P and found that the serine residue at position 308 (S308) within P protein resembles a PLK1 phosphorylation site (Fig. 5A). Mutation of S308 should thus result in a P with higher activity in the mini-genome system that is insensitive to PLK1. As shown in Fig. 5B, mutating S to A at 308 (P-S308A) increased the mini-genome activity when compared with wild type P. Mutating S304 and S313, two other S residues close to S308, had no effect on the mini-genome activity (supplemental Fig. S4A and S4B). In addition, PLK1 inhibitor BI 2536 treatment did not affect P-S308A mini-genome activity (Fig. 5C). Furthermore, overexpression of PLK1 had no effect on the activity of the mini-genome system using P-S308A (Fig. 5D), indicating that S308 is a PLK1 phosphorylation target. To exclude the possibility that the mutation at S308 caused a conformational change in P such that it can no long be bound by PLK1, we examined the interaction between PLK1 and P-S308A. As shown in Fig. 5E, P-S308A co-precipitated with PLK1 and vice versa.

To confirm that PLK1 phosphorylates P protein at S308, we carried out an *in vitro* kinase assay. P proteins were purified from HeLa cells transfected with plasmids encoding either P or P-S308A by affinity chromatography using anti-P antibody conjugated agarose gel. Purified P was then incubated with PLK1 purchased from a commercial vendor. As shown in Fig. 5F, wild type P was phosphorylated by PLK1, while P-S308A was not, indicating that S308 within P protein is the phosphorylation site of PLK1.

### PLK1 targets S308 of P in infected cells

**Figure 6:**
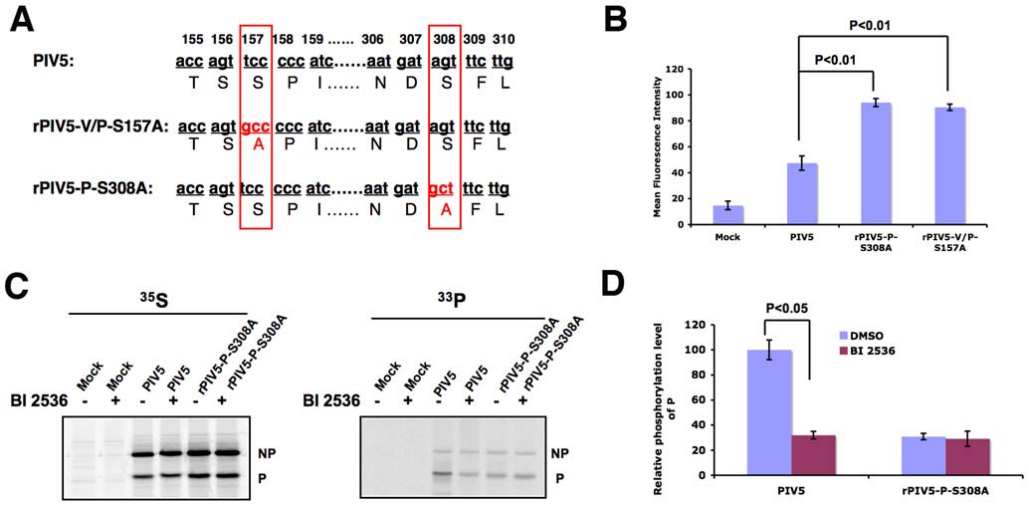
Effect of mutating S to A at position 308 in recombinant virus. (A). Sequences of PIV5, rPIV5-V/P-S157A and rPIV5-P-S308A at regions around residue 157 and 308 of the P protein. The entire genome of the mutant viruses was sequenced and no mutation was observed except in the designated site (S157A or S308A). (B). Viral gene expression from PIV5, rPIV5-V/P-S157A and rPIV5-P-S308A-infected cells. HeLa cells were infected with PIV5, rPIV5-V/P-S157A, or rPIV5-P-S308A. At 16 hours post infection, the infected cells were processed for flow cytometry using anti-HN antibody as described in Experimental Procedures. Average mean fluorescence intensity of infected cells +/− SEM is graphed. (C). Effects of PLK1 inhibitor on phosphorylation of P of rPIV5-P-S308A. Cells were mock-infected or infected with PIV5 or rPIV5-P-S308A. At 18–20 hours post infection, the cells were metabolically labeled with ^35^S-Cys/Met or ^33^P-orthophosphate in the presence of 1 µM PLK1 inhibitor BI 2536 or DMSO as described in Fig. 4A. The cells were lysed and immunoprecipitated with ant-P Pk antibody. (D). Quantification of effects of BI 2536 on phosphorylation of P of rPIV5-P-S308A. Three individual experiments as described in (C) were quantified. The average ratio of phosphorylated P from the ^33^P-labeling experiment to total amount of P as ^35^S-labeled P in PIV5 infected cells treated with DMSO was set to 100 and the others are normalized to the ratio. The average of relative phosphorylation level of P +/− SEM is shown.

Previously, mutations affecting P phosphorylation were identified and tested using *in vitro* or mini-genome assays. However, the results were not substantiated when the sites are incorporated into recombinant viruses. Thus, to determine whether phosphorylation of P affects viral gene expression, it is essential to examine the effects of putative mutations in the context of virus infection. Using a PIV5 reverse genetics system, we generated two recombinant viruses encoding mutant P proteins: rPIV5-V/P-S157A, which has a single amino acid change in the PLK1 binding site; and rPIV5-P-S308A, which has a single amino acid residue change at 308 at the PLK1 phosphorylation site (Fig. 6A). The mutant viruses grew to normal titers and formed plaques similarly to wt PIV5 (data not shown). Viral gene expression levels from cells infected with the viruses were examined by flow cytometry using anti-HN antibody. As shown in Fig. 6B, cells infected by rPIV5-V/P-S157A or rPIV5-P-S308A expressed higher levels of HN than PIV5-infected cells, consistent with previous observations.

To investigate whether PLK1 phosphorylates the P protein at S308 in infected cells, we performed ^33^P-orthophosphate labeling of infected cells with or without BI 2536 treatment similar to the experiment described in Fig. 4A. As shown in Fig. 6C and 6D, the P protein in cells infected by rPIV5-P-S308A had lower levels of phosphorylation than that in PIV5-infected cells. Phosphorylation of P-S308A in infected cells was not sensitive to BI 2536 treatment while phosphorylation level of wild type P was reduced by PLK1 inhibitor BI 2536, indicating that S308 is a phosphorylation target for PLK1 in infected cells. These results mirror those presented in Fig. 4 using rPIV5-Cpi+, whose P protein encodes Ser at position 308 but no longer binds PLK1 due to the S157F mutation, underscoring the importance of both binding and phosphorylation of P by PLK1.

### Induction of apoptosis and cytokine expression by mutant viruses

**Figure 7:**
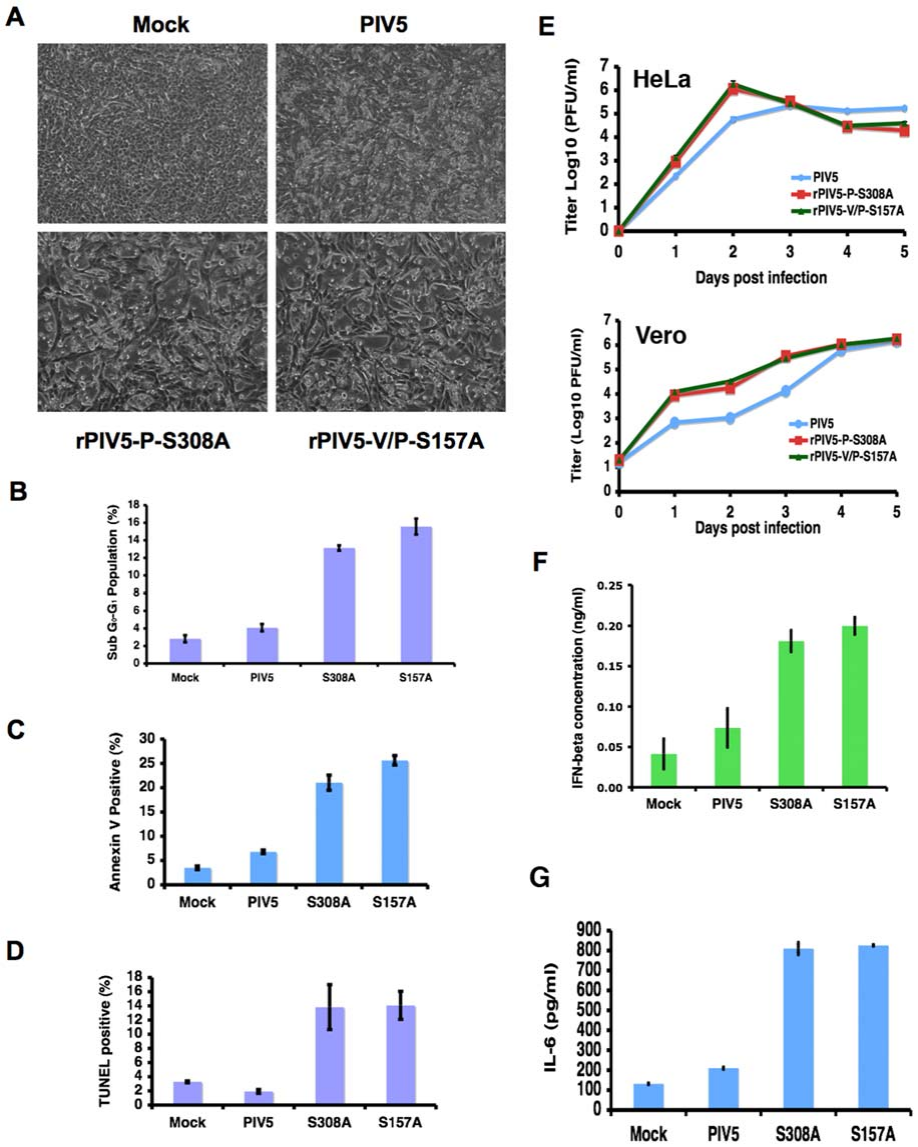
Induction of apoptosis and cytokine expression by mutant viruses. (A) Induction of cytopathic effect by virus infection. MDBK cells were infected with PIV5, rPIV5-V/P-S157A, or rPIV5-P-S308A. At 48 hours post infection, the infected cells were photographed using microscope (Nikon ECLIPSE TE300, Japan). (B) Propidium iodine (PI) staining. MDBK cells were infected and collected 2 days post infection as above. The cells were stained with PI and cellular DNA profiles were examined using flow cytometry. Percentages of sub G_0_-G_1_ population, which is considered apoptotic, are graphed. S157A, rPIV5-V/P-S157A; S308A, rPIV5-P-S308A. (C). Annexin V staining. The infected cells were stained with FITC-labeled annexin V and measured using a flow cytometer. Annexin V, which binds to phosphatidylserine (PS), is an indication of cells undergoing apoptosis when it is present on cell surface. Percentages of annexin V positive cells are graphed. (D) TUNEL assay. The infected cells were subjected to a TUNEL assay as described in Experimental Procedures. TUNEL positive cells were measured using a flow cytometer and percentages of TUNEL positive cells are graphed. (E). Growth of recombinant viruses. HeLa cells or Vero cells were infected with viruses at 0.01 MOI and media of infected cells were collected at indicated time points. Titers of viruses were determined using plaque assay. (F). Induction of IFN-β. HeLa cells were infected and levels of IFN-β were measured using ELISA at 2 days post infection. (G). Induction of IL-6. HeLa cells were infected and levels of IL-6 were measured using ELISA at 2 days post infection. Error bars are standard deviation of mean.

It has been reported that rPIV5-CPI− virus, which contains a mutation at S157, causes increased cell death and cytokine expression in addition to the increased levels of viral gene expression. To investigate whether recombinant PIV5 containing these mutations described above cause increased cell death, MDBK cells were infected and photographed. As shown in Fig. 7A, the viruses caused increased cell death. To further investigate the nature of the cell death, apoptosis assays were performed on the cells infected with mock, wild type, rPIV5-P-S308A or rPIV5-V/P-S157A. Both mutant viruses induced increased apoptosis in the infected cells using propidium iodine staining, which detects the sub-G_0_-G_1_ population, annexin V staining, which detects phosphatidylserine (PS) on the surface, and TUNEL assay, which detects nicked DNA (Fig. 7B, C and D). To examine growth of recombinant viruses in cultured cells, HeLa cells and Vero cells were infected with recombinant viruses at low MOI and virus titers in media of infected cells were determined at various times post-infection by plaque assay. Interestingly, while rPIV5-V/P-S157A and rPIV5-P-S308A grew fast than wild type initially as expected since the mutant viruses had higher levels of viral gene expression, their growth was reduced at later time points in HeLa cells, which are capable of producing and responding to interferon. This drop of growth was not observed in Vero cells, which do not produce interferon (Fig. 7E), indicating that interferon may play a role in growth of the viruses. To investigate induction of cytokines by the mutant viruses, HeLa cells were infected and media from infected cells were collected. Amounts of IFN-β, an anti-viral cytokine, and IL-6, a proinflammatory cytokine, were measured using ELISA. Expression levels of both cytokines were elevated in rPIV5-P-S308A and rPIV5-V/P-S157A-infected cells compared to mock or wild type virus-infected cells (Fig. 7F and G).

## Discussion

Our studies present the first evidence that phosphorylation of a paramyxovirus P protein directly regulates viral gene expression. Mutating phosphorylation sites within the P protein of SeV such that P phosphorylation is reduced by more than 90% in transfected cells does not affect its activity in transcription, either in a mini-genome system or in recombinant virus [5]. Similarly, mutation of the major phosphorylation sites within the P protein of RSV results in a mutant P whose level of phosphorylation is reduced by more than 90%. Yet, when these mutations are introduced into the RSV genome by reverse genetics, expression levels of the viral genes are not adversely affected. These results seemingly suggest that phosphorylation of the P proteins of paramyxoviruses does not have a role in viral gene expression. However, it is possible that the critical phosphorylation sites within the P proteins have not been identified in these earlier studies. While it is true that some of the phosphorylation sites may be superfluous and may not have a role in viral gene expression, it is difficult to imagine that none of the phosphorylation sites within a heavily phosphorylated P protein have any role in its function. Even though mutation of known phosphorylation sites reduces phosphorylation of SeV and RSV P by 90%, it is possible that the remaining residues that are phosphorylated (even though they only count for about 10% of total phosphorylation) are critical for viral gene expression. In this work, we have identified a host kinase, PLK1, which phosphorylates the P protein and mapped both its binding (SSP motif at S157) and phosphorylation (S308) sites within P. Further, we demonstrated that this site (S308) is important in regulating viral gene expression using a recombinant virus. To the best of our knowledge, this is the first report of a host kinase that directly phosphorylates the P protein and regulates viral gene expression of a paramyxovirus. Interestingly, PLK1 phosphorylation down-regulates viral gene expression, contrary to our expectation that phosphorylation may be essential for viral gene expression. It is possible that additional phosphorylation sites within the P protein may play an essential role in the regulation of viral gene expression. Previously, we have found that AKT1, a serine/threonine kinase, plays a critical role in viral gene expression [28]. However, direct interaction between P and AKT1 as well as phosphorylation site of AKT within the P protein has not been reported. Further studies of phosphorylation of the P protein will be needed to identify possible phosphorylation sites within P that are critical for viral gene expression.

**Figure 8:**
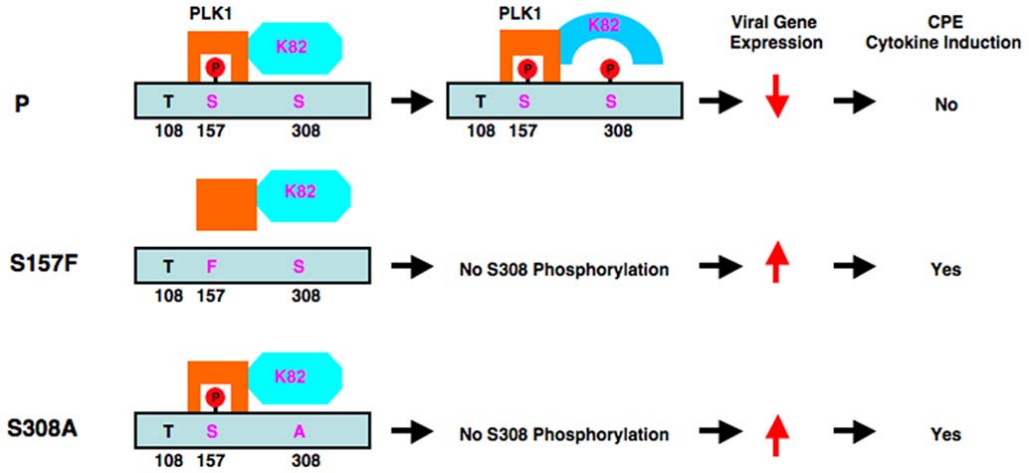
A working model for regulation of PIV5 viral gene expression by PLK1. For wild type PIV5, PLK1 binds to SSP motif (156–158) within the P protein, and then phosphorylates the P protein at S308, resulting in inhibition of PIV5 gene expression. The reduced viral gene expression supports efficient virus replication, but enables virus to avoid the induction of cell death and cytokine expression. For rPIV5-CPI+, SSP is mutated to SFP, preventing the binding of P to PLK1 and, thus, no phosphorylation of S308 by PLK1. As a result, viral gene expression is higher than that of PIV5, and cell death as well as cytokine production is induced, resulting in limiting replication and spread of virus. However, when PLK1 is over expressed, PLK1 can interact with S157F weakly through STP centered at T108 and reduces P-S157F mini-genome expression moderately. For rPIV5-P-S308A, while the SSP motif is intact and P-S308A still binds to PLK1; however, there is no phosphorylation of S308. This mutant virus, similar to rPIV5-CPI+, shows elevated viral gene expression and increased induction of cell death.

Previously, we showed that S157 was phosphorylated and contributes to the phenotypes of rPIV5-CPI−. However, it was formally possible that the V protein, which also contains the S157 mutation, contributed to the CPI− phenotypes since V and P are identical in the first 164 amino acid residues. In this work, we have identified a single amino acid residue (S308) within the P protein as a PLK1 phosphorylation site. Mutating this phosphorylation site has the same effect on viral gene expression as the CPI− P mutations, further confirming our previous report that the increased gene expression from rPIV5-CPI− is due to a P protein that is more efficient in facilitating viral RNA synthesis since the S308A mutation is within the unique P sequences while the V remains intact. It may seem counterintuitive that a virus would down-regulate its own gene expression by maintaining binding and phosphorylation sites for PLK1. However, higher PIV5 gene expression is associated with the induction of cytokine expression and cell death, which in turn limits virus replication and spread. Therefore, viruses that down-regulate their gene expression via PLK1 phosphorylation of P would have advantages in viral transmission over viruses with defective PLK1 binding or phosphorylation sites (Fig. 8). Since increased cell death and cytokine expression induced by virus infection limit virus replication, it is possible that targeting PLK1 can enhance host innate immune responses, leading to a novel strategy to control virus infection. The lower viral gene expression after PLK1 phosphorylation may also favor persistent viral infection. Interestingly, about half the strains of PIV5 have an S residue at position 157 [15].

PLK1 can phosphorylate proteins with a PLK1 binding domain as well as a proteins associated with PLK1-binding proteins. Thus, it is possible that PLK1 down-regulates viral gene expression through phosphorylation of a P-binding protein. However, the S308A mutant, which still binds PLK1, has the same viral gene expression phenotype as PLK1 binding site (S157) mutants, indicating that P is not merely a scaffold for PLK1 to down-regulate viral gene expression, but is the target of PLK1 itself. The exact mechanism of how the phosphorylation by PLK1 at residue S308 affects the function of P remains to be determined. The P protein does not have a catalytic domain but it plays an essential regulatory role in viral gene synthesis. P is known to interact with viral proteins NP and L. Binding to and phosphorylation by PLK1 do not appear to have an impact on the interactions between P and NP or L. Previously, it has been reported that rPIV5-CPI− has an increased viral RNA genome synthesis while viral mRNA transcription is not affected [21]. It is possible that phosphorylation at S308 changes the conformation of P to make it less efficient in facilitating viral RNA genome synthesis. It is possible that phosphorylated P (at 308) binds to RNA better and increases the rate of RNA synthesis. However, we cannot rule out the possibility that phosphorylation of P at S308 may affect its ability to interact with a yet-to-be identified host protein, which in turn regulates viral gene expression.

A role for PLK1 in infection by other viruses has been reported recently. Expression of human T-lymphotropic virus type-I protein p30 reduces expression as well as phosphorylation of PLK1 [29] while PLK1 is up-regulated in CD4+ T cells from rhesus macaques infected with simian immunodeficiency virus [30] as well as in cells expressing E6/E7 of human papillomavirus [31]. PLK1 can directly phosphorylate pp65, a dispensable gene of human cytomegalovirus [32]. However, none of these studies has demonstrated a direct role of PLK1 in viral gene expression and the biological consequence of these studies are not clear. Recently, Ludlow et al reported that the V protein of NiV contains a PLK1 binding site within the shared region of the NiV P protein and that the PLK1 binding site is also involved in interacting with Stat1 protein, a key protein in IFN signaling [33]. However, the biological consequence of this PLK1 interaction is not clear; since Nipah virus is a biosafety level 4 pathogen, examining the role of PLK1 in the context of Nipah virus infection is difficult. Ours is the first report of PLK1 playing a direct role in virus replication.

The PLK1 binding site within the P protein of PIV5 is within the shared region of the V and P protein; thus, the PIV5 V should bind PLK1 as well. It is well known that the V protein blocks interferon signaling by causing degradation of Stat1. However, it is unlikely this PLK1 binding site is involved in interferon signaling since the mutations at the S157 residue have no effect on IFN signaling [19]. It is possible that PLK1 plays additional roles in viral life cycle. For instance, PIV5 V protein is known to slow down the cell cycle [34], so it is tempting to hypothesize that PLK1 is involved in the regulation of cell cycle by the V protein.

A cursory examination of the P proteins of paramyxoviruses indicates that most contain SSP (or STP) sequence motifs; some of the P proteins have more than one SSP sequence motif. For instance, NiV P has two SSP sequences. However, it is not known whether those S/T residues are phosphorylated. Since efficient PLK1 binding requires the middle S/T residue to be phosphorylated [25], it is not clear whether these SSP sequences are truly SSP motifs and bind PLK1. It is possible that the role of PLK1 in viral gene expression is universal for all paramyxoviruses. On the other hand, it is equally possible that PLK1 plays different roles for different paramyxoviruses. These questions warrant further investigation.

## Materials and Methods

### Plasmids, viruses, and cells

Plasmids expressing P mutants, Pcpi+, P-S157A and P-S308A were made from a copy of P of W3A strain in the pCAGGS vector [35]. The human PLK1 gene was obtained from Open Biosystems (AL, USA). A Flag tag was added to the N-terminus of PLK1 and cloned into pCAGGS vector. Kinase-deficient PLK1 (Flag-PLK1-K82M) was constructed by PCR using Flag-PLK1 as the template and pfx50™ DNA polymerase (Invitrogen, 12355-012), and confirmed by sequencing. Plasmids containing full-length genome for rPIV5-V/P-S157A and rPIV5-P-S308A viruses were made similarly to that of rPIV5-CPI+ as described before [21] and confirmed by sequencing. Plasmids used in PIV5 mini-genome system have been described before [21],[36]. The details of plasmid construction and sequences of the plasmids are available on request. rPIV5-V/P-S157A and rPIV5-P-S308A were rescued from the plasmids containing their respectively full-length genome as described before [21]. PIV5, rPIV5-CPI+ and rPIV5-RL viruses used in this study were described as before [21]. HeLa and MDBK cells were grown in Dulbecco modified Eagle medium (DMEM) (Invitrogen, USA) containing 10% fetal bovine serum (FBS), 100 IU/ml penicillin, and 100 µg/ml streptomycin. BSR T7 cells were grown in the same media as HeLa plus 10% tryptose phosphate broth (TPB) and 400 µg/ml G418. All cell lines were incubated at 37°C in 5% CO_2_. The growth medium for infected cells contains only 2% FBS. Virus titers were determined using BHK cells as described before [37],[38].

### Viral genome sequencing

MDBK cells were infected with mutant virus stock with MOI of 1. At two days post infection, viral RNA was extracted from the media by QIAmp viral RNA mini kit (Qiagen, CA) following the protocol provided by the manufacture. The viral RNA was reverse transcribed with random primer (Superscript III first-Strand Synthesis System, Invotrogen). 12 pairs of oligomers were used for PCR and 30 oligomers were used for sequencing. The terminal sequences of viral genome were obtained by Rapid Amplification of cDNA Ends (RACE) as described before [39]. Briefly, to sequence 3′ leader sequence, viral RNA was ligated with an adaptor JX129 then reverse transcribed by JX130 (complementary to JX129). The cDNA were amplified by the JX130–JX131 pair and then sequenced. To sequence 5′ trailer sequence, the viral RNA was firstly reverse transcribed using JX133. The cDNA products were purified and ligated to JX129. The ligation product was amplified with JX130–JX132. The PCR products were purified, sequenced and compared with wide type PIV5 genome using BLAST. Sequences of all oligomers used for PCR or sequencing are available on request.

### Immunoprecipitation

To detect interactions between P and endogenous PLK1 in infected cells, HeLa cells in 10 cm plates were infected with mock, PIV5 or rPIV5-CPI+ at multiplicity of infection (MOI) of 3 for 12 h then treated with 100 ng/ml Nocodazole, which is known to increase expression levels of PLK1 by arresting cells into M phase when PLK1 expression levels are the highest [40]–[42]. After10–12 h incubation with Nocodazole, the cells were lysed by Whole Cell Extraction Buffer (WCEB: 60 mM Tris-HCl pH 6.8, 40% glycerol, 4%SDS, 3% dithiothreitol (DTT), and few grains of bromophenol blue) containing protease inhibitor cocktail as described before [21],[43]. The same amount of the lysates were pre-cleared with 40 µl protein G sepharose beads for 1 h at 4°C. The cell lysate were then incubated with P antibody and 30 µl protein G sepharose beads for 2–3 h at 4°C. The precipitated proteins were resolved by 15% sodium dodecyl sulfate polyacrylamide gel electrophoresis (SDS-PAGE), and transferred onto PVDF membrane (Milipore, MA). Mouse anti-PLK1 antibody (Santa Cruz Biothechnology) was used to detect PLK1 by immunoblotting as described before [21],[43]. To examine whether PLK1 interacts with P directly, not through other viral proteins, BSR T7 cells in 6 cm plates were transfected with pCAGGS vector, pCAGGS-P, or pCAGGS-Pcpi+ with pCAGGS-Flag-PLK1 using Plus and Lipofectamine reagents following the protocol from manufacturer (Invitrogen). The transfected cells were incubated with DMEM deficient of Cys/Met for 30 minutes and then labeled with DMEM containing ^35^S-Cys/Met Promix (Amersham Life Sciences) (100 µCi/ml) for 3 h. The cells were lysed in RIPA buffer (20 mM Tris-HCl pH 7.4, 150 mM NaCl, 0.2% Triton-X100, 0.1% SDS, 5 mM Iodoacetanide) containing protease inhibitor cocktail. Pk antibody or Flag antibody together with Protein G sepharose beads were used for immunoprecipitation. The precipitated proteins were washed three times with RIPA buffer containing 0.3 M NaCl and once with RIPA buffer containing 0.15 M NaCl. After washing, the precipitated proteins were resolved by 15% SDS-PAGE, and proteins were detected by autoradiography using Storm Phosphorimager (Molecular Dynamics Inc., Sunnyvale, CA).

### PLK1 inhibitors

BI 2536, a highly selective PLK1 inhibitor was purchased from Axon Company (The Netherlands) [41]. The compound was dissolved in dimethyl sulfoxide (DMSO). To study the effect of PLK1 inhibitor on rPIV5-RL gene expression, HeLa cells or BSR T7 cells at about 90% confluence were infected with rPIV5-RL at a MOI of 1 and incubated with the media containing 2% FBS and PLK1 inhibitor. *Renilla* luciferase activity was examined by luminometer using *Renilla* luciferase assay kit (Promega) at 16 to 20 hours post infection. Similarly, BI 2536 was tested in the mini-genome system. GW843682 (Sigma), a PLK1 inhibitor, which inhibits PLK1 and weakly inhibits PLK3 (which has similar function as PLK1) [44], was also used.

### Phosphorylation of P

To examine phosphorylation of P, infected cells in 6 cm plates were incubated in the medium lacking sodium phosphate for 30 minutes and then labeled with medium containing ^33^P-orthophosphate (100 µCi) in the present of DMSO or 1 µM BI 2536 for 4 hour. The cells were then lysed, immunoprecipitated using Pk antibody, and resolved in 15% SDS-PAGE and quantified as before.

### PIV5 mini-genome system and dual luciferase assay

PIV5 mini-genome system used in this study was described before [21], except that negative controls contained no P plasmid, instead of no L plasmid. BSR T7 cells in 24-well plates were transfected with plasmids expressing pSMG-RL (0.2 µg), NP (0.2 µg), P (0.01–0.16 µg), L (0.3 µg) as well as a firefly luciferase gene (FF-Luc) under control of a T7 promoter using Plus and Lipofectamine reagents (Invitrogen). GFP plasmid was used as a control to keep the total amount of transfected plasmids the same. For transfection of Flag-PLK1 or Flag-PLK1-K82M together with the mini-genome system composed of P, P-S157A orP-S308A, P or P mutant plasmids were used at 0.02 µg. At 18–22 hrs post transfection, the cells were lysed with 100 µl 1× passive lysis buffer (Promega). 10 µl lysate from each well were used for dual luciferase assay by dual luciferase assay kit (Promega). Ratios of R-Luc to FF-Luc activity are normalized as relative activity unit. Six replicates of each sample were used for statistical analysis. An aliquot of the cell lysate from mini-genome system was mixed with equal volume of 2× protein lysis buffer as described before. Samples were resolved in 10% SDS-PAGE and transferred onto PVDF membrane. Mouse anti-NP and mouse anti-P (Pk antibody) were used together for immunoblotting. Anti-Flag was used to detect the amount of input of Flag-PLK1 or Flag-PLK1-K82M.

### *In vitro* kinase assay

For PLK1 *in vitro* kinase assay, HeLa cells in 10 cm plates were transfected with pCAGGS-P-wt or pCAGGS-P-S308A. At 24 hours post transfection, anti-V5 agarose gel, which has Pk antibody conjugated to the agarose beads, was used to purify P-wt or P-S308A using immunoprecipitation. Similar amounts of P-wt and P-S308A were used for PLK1 (Cell Signal, MA) *in vitro* kinase assay with kinase buffer (25mMHEPES, 25 mM beta-glycerophosphate, 25 mM MgCl_2_, 2 mM dithiothreitole, and 0.1 mN NaVO_3_). Half of the reaction mixture was used for SDS-PAGE followed by Coomassie blue staining to measure the amount of input P-wt and P-S308A, and the other half of the mixture was incubated with 10 µCi γ-^32^P-ATP (Amersham Biosciences) for 1 h at room temperature in a total volume of 30 ul containing 100 ng PLK1. Reactions were terminated by addition of the same volume of 2× SDS loading buffer. Proteins were separated by 15% SDS-PAGE and phosphorylation was detected with a Storm PhosphorImager (Molecular Dynamics).

### Flow cytometry

To detect different levels of viral gene expression in Mock, PIV5, rPIV5-P-S308A, and rPIV5-V/P-S157A infected cells, flow cytometry was carried out as previously descrbed [21]. Briefly, HeLa cells at about 90% confluence were mock infected or PIV5, rPIV5-V/P-S157A, or rPIV5-P-S308A infected at MOI of 3. The infected cells were collected at 16 h hpi and fixed with 0.5% formaldyhyde for 2 h at 4°C. After centrifugation and removal of the supernatant, the cells were re-suspended in 500 µl solution of FBS-DMEM (50∶50), then permeabilized in 70% ethanol overnight at 4°C. The cells were washed with phosphate buffered saline without Mg^2+^ and Ca^2+^ (PBS-) and incubated with mouse anti-HN in PBS- with 10% FBS for 30 min at room temperature. After washing with PBS-, the cells were further stained with FITC-conjugated goat anti-mouse antibody in the dark and washed with PBS-. The fluorescence intensity was measured using a flow cytometer (FC500).

### Apoptosis assays

To detect apoptotic cells, annexin-V staining, propidium iodide (PI) staining, and terminal deoxynucletidyltransferase-mediated dUTP-FITC nick end labeling (TUNEL) assay were performed as previously described [45],[46]. Briefly, MDBK cells in 6-well plates were infected with Mock, PIV5, rPIV50P-S308A or rPIV5-V/P-S157A with MOI of 5 for 48 h. The floating cells were harvested together with the trypsinized monolayer cells. For annexin-V staining, the cells were washed with cold PBS- and incubated with FITC-labeled annexin-V (Annexin-V-FLIOS, Roche Diagnostics Corp. Mannheim, Germany) for 15 min at room temperature. 10,000 cells were examined for fluorescence by a flow cytometer FC500. For PI staining, the collected cells were fixed and permeabilized as before. The permeabilized cells were washed and incubated with 100 µl Pk antibody (1∶100 dilution in PBS-1% BSA) for 30 min at room temperature. After washing, the cells were incubated with 100 µl FITC-labeled anti-mouse antibody (1∶1,000 in PBS-1%BSA). The cells were finally incubated with 500 µl of PI solution (50 µg/ml) (Sigma-Aldrich). The stained cells were examined with a flow cytometer. For TUNEL assay, the permeabilized cells were incubated with 25 µl of TUNEL reaction mixture (Roche Diagnostics Corp. Mannheim, Germany) for 3 h in the dark at 37°C. After washing, the cells were incubated with 100 µl Pk antibody (1∶100 dilution in PBS-1% BSA) then 100 µl Phycoerythrin-labeled anti-mouse antibody (1∶100 dilution in PBS-1%BSA) and analyzed with FC500.

### Enzyme-linked immunosorbent assay (ELISA) for IFN-β and IL-6

HeLa cells infected with mock, PIV5, rPIV5-P-S308A, rPIV5-V/P-S157A were infected with MOI of 5. The media were collected at 48 h post infection and centrifuged to remove cell debris. 50 µl cleared media or IFN-β standard in duplicate were used for IFN-β ELISA by using human IFN-β ELISA kit (PBL InterferonSource, NJ). For IL-6 production, the same media samples were sent to The General Clinical Research Center (GCRC) at Pennsylvania State University to measure IL-6 as described in Lin et al [47].

## Supporting Information

Figure S1 Interaction between PLK1 and Pcpi+. The cells were transfected as in Fig. 1B. However, the cells were not metabolically labeled and immunoprecipitated as in Fig. 1B. The immunoprecipitated peptides were resolved in SDS-PAGE and subjected to immunoblotting using antibodies against P or PLK1.
(0.84 MB TIF)

Figure S2 Effect of PLK1 inhibitor GW843682 on PIV5 gene expression. GW843682 (Sigma), a PLK1 inhibitor, was examined. HeLa cells in 24-well plates were infected with rPIV5-RL at MOI of 1 and incubated with GW843682 at 37°C for 16 to 20 hours. Luciferase activities from the cells were measured as described in Fig. 2A. The average of relative luciferase activity +/− SEM is shown.
(0.74 MB TIF)

Figure S3 Effects of P with S changed to A at position 157 (P-S157A). (A). Interaction between P-S157A and PLK1. A plasmid encoding Flag-PLK1 was transfected into cells with a plasmid encoding P or P-S157A. The cells were metabolically labeled and immunoprecipitated with anti-P or anti-Flag. (B). Effects of PLK1 overexpression on the mini-genome system using P-S157A. Plasmids encoding pCAGGS-Flag-PLK1 at various concentrations were transfected along with the mini-genome system. An aliquot of cell lysate was used for immunoblotting to detect expression levels of Flag-PLK1. The average of relative luciferase activity +/− SEM is shown.
(2.43 MB TIF)

Figure S4 Effects of mutating serine residues close to the serine residue at position 308 of P on mini-genome gene expression. (A). Comparison of the mini-genome activity of P to that of P with a S to A change at position 304. (B). Comparison of the mini-genome activity of P to that of P with a S to A change at position 313. The average of relative luciferase activity +/− SEM is shown.
(1.26 MB TIF)
