# Supplementary figures and images for: PLK1 Down-Regulates Parainfluenza Virus 5 Gene Expression
Source: PLoS Pathog. 2009 Jul 24;5(7):e1000525. doi: 10.1371/journal.ppat.1000525 (PMC2709441; doi:10.1371/journal.ppat.1000525)

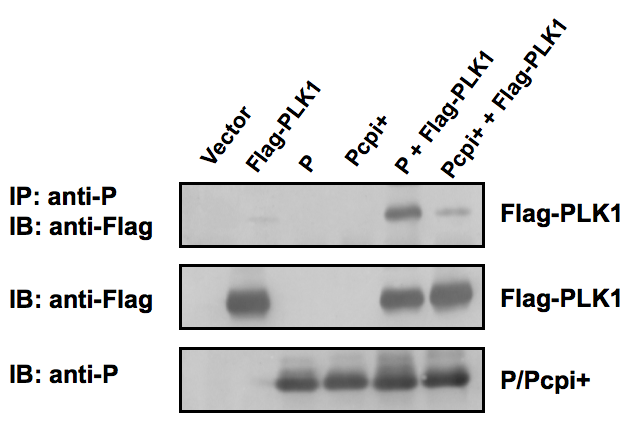

Supplement: Figure S1 — Interaction between PLK1 and Pcpi+. The cells were transfected as in Fig. 1B. However, the cells were not metabolically labeled and immunoprecipitated as in Fig. 1B. The immunoprecipitated peptides were resolved in SDS-PAGE and subjected to immunoblotting using antibodies against P or PLK1. (0.84 MB TIF) [file ppat.1000525.s001.tif]

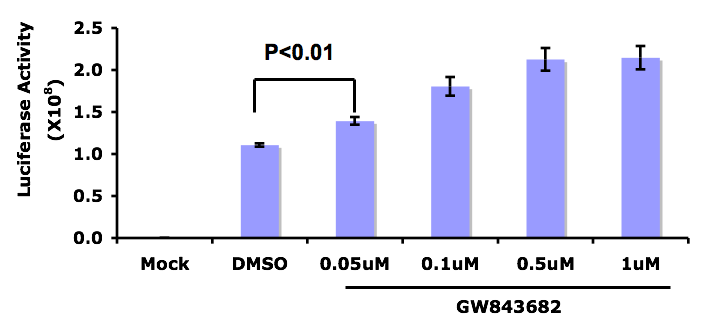

Supplement: Figure S2 — Effect of PLK1 inhibitor GW843682 on PIV5 gene expression. GW843682 (Sigma), a PLK1 inhibitor, was examined. HeLa cells in 24-well plates were infected with rPIV5-RL at MOI of 1 and incubated with GW843682 at 37°C for 16 to 20 hours. Luciferase activities from the cells were measured as described in Fig. 2A. The average of relative luciferase activity +/− SEM is shown. (0.74 MB TIF) [file ppat.1000525.s002.tif]

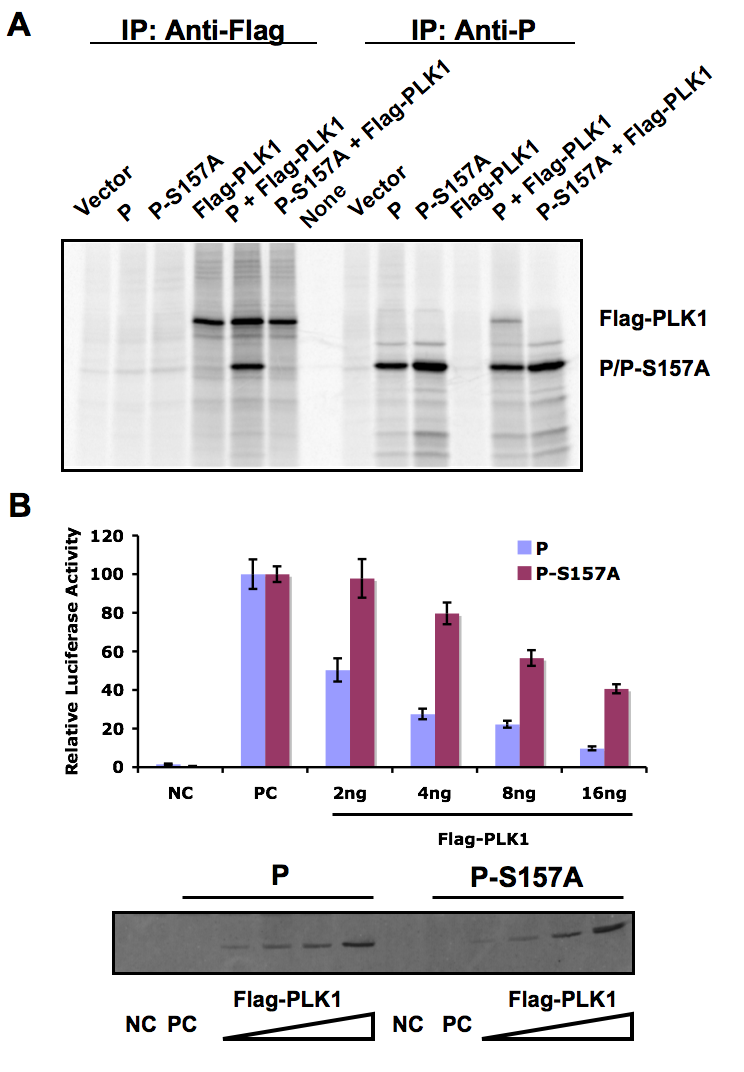

Supplement: Figure S3 — Effects of P with S changed to A at position 157 (P-S157A). (A). Interaction between P-S157A and PLK1. A plasmid encoding Flag-PLK1 was transfected into cells with a plasmid encoding P or P-S157A. The cells were metabolically labeled and immunoprecipitated with anti-P or anti-Flag. (B). Effects of PLK1 overexpression on the mini-genome system using P-S157A. Plasmids encoding pCAGGS-Flag-PLK1 at various concentrations were transfected along with the mini-genome system. An aliquot of cell lysate was used for immunoblotting to detect expression levels of Flag-PLK1. The average of relative luciferase activity +/− SEM is shown. (2.43 MB TIF) [file ppat.1000525.s003.tif]

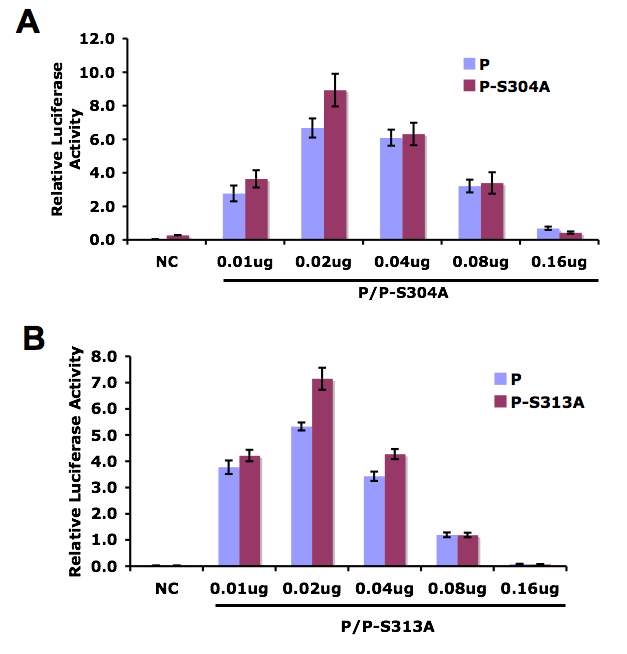

Supplement: Figure S4 — Effects of mutating serine residues close to the serine residue at position 308 of P on mini-genome gene expression. (A). Comparison of the mini-genome activity of P to that of P with a S to A change at position 304. (B). Comparison of the mini-genome activity of P to that of P with a S to A change at position 313. The average of relative luciferase activity +/− SEM is shown. (1.26 MB TIF) [file ppat.1000525.s004.tif]
